# Supplementary material for: Critical role of electrons in the short lifetime of blue OLEDs
Source: Nat Commun. 2023 Nov 18;14:7508. doi: 10.1038/s41467-023-43408-7 (PMC10657374; doi:10.1038/s41467-023-43408-7)
Supplement: Supplementary file 1 — Supplementary Information [file 41467_2023_43408_MOESM1_ESM.pdf]

Supplementary Information

for

Critical role of electrons in the short lifetime of blue OLEDs

Jaewook Kim[1†], Joonghyuk Kim[2†], Yongjun Kim[1], Youngmok Son[2], Youngsik Shin[2], Hye Jin Bae[2], Ji Whan Kim[2], Sungho Nam[2], Yongsik Jung[2], Hyeonsu Kim[1], Sungwoo Kang[1‡], Yoonsoo Jung[1], Kyunghoon Lee[1], Hyeonho Choi[2*], and Woo Youn Kim[1*]

[1] *Department of Chemistry, KAIST, 291 Daehak-ro, Yuseong-gu, Daejeon 34141, Republic of Korea*

[2] *Samsung Advanced Institute of Technology, Samsung Electronics Co., Ltd., 130 Samsung-ro, Suwon-si, Gyeonggi-do 16678, Republic of Korea*

[‡]*Current address: Innovation Center, Samsung Electronics Co., Ltd., 1 Samsungjeonja-ro, Hwasung-si, Gyeonggi-do, 18448, Republic of Korea*

[†]Jaewook Kim and Joonghyuk Kim contributed equally to this work.

[*] Corresponding authors: Hyeonho Choi (hono.choi@samsung.com) and Woo Youn Kim (wooyoun@kaist.ac.kr)

Supplementary Figures

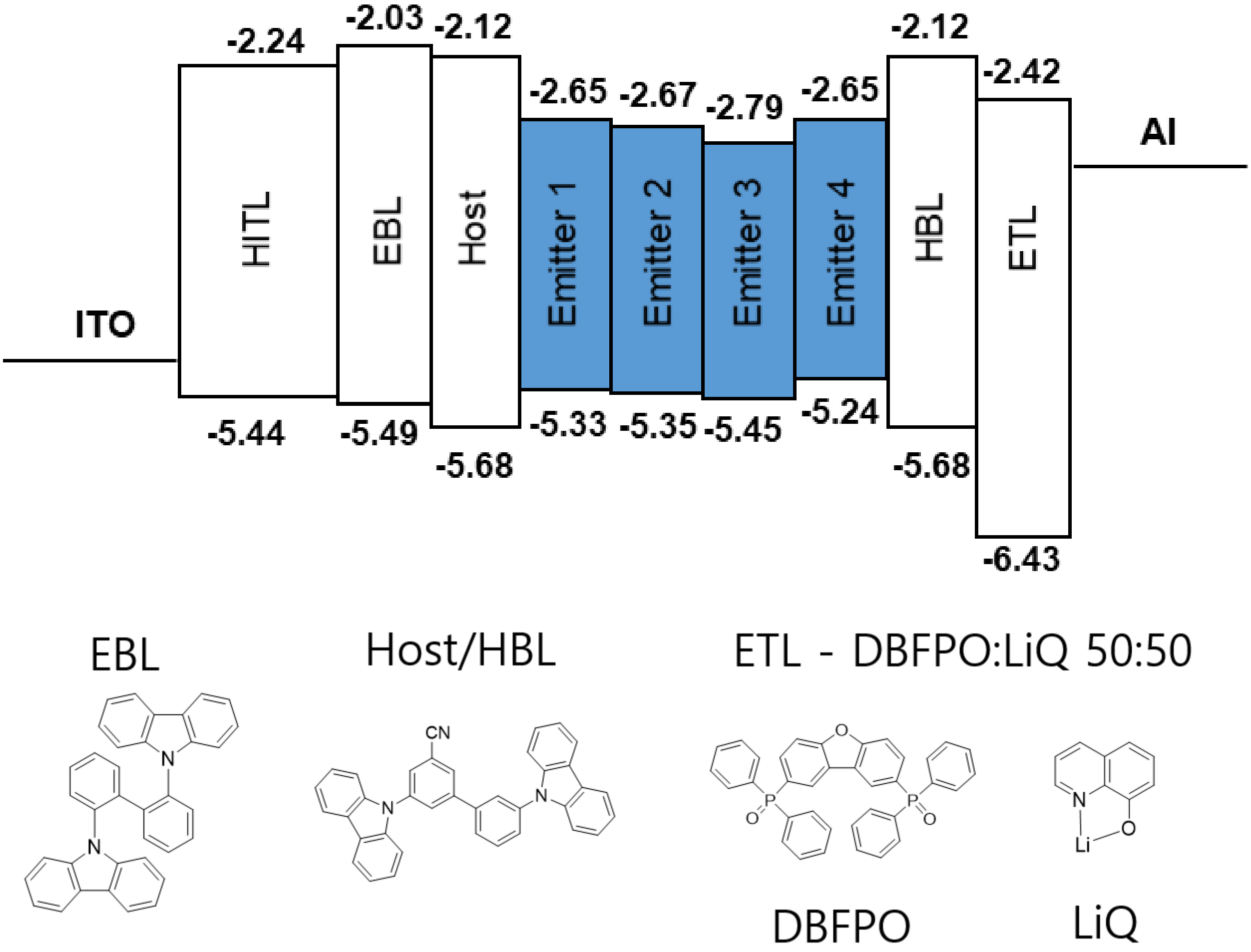

Supplementary Figure S1. Schematic diagram of the configuration of the electroluminescence devices (whole devices) and their component materials.

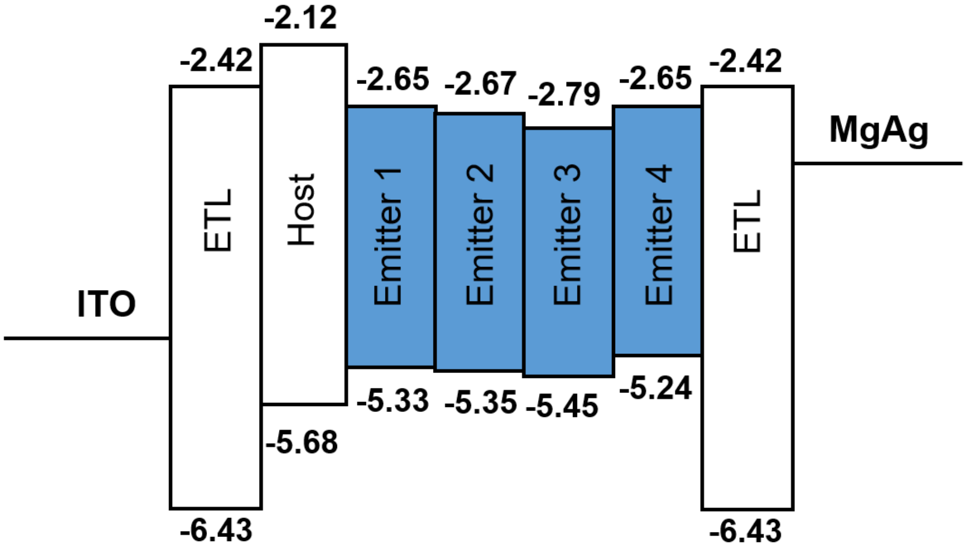

Supplementary Figure S2. Schematic diagram of the configuration of the electron-only devices.

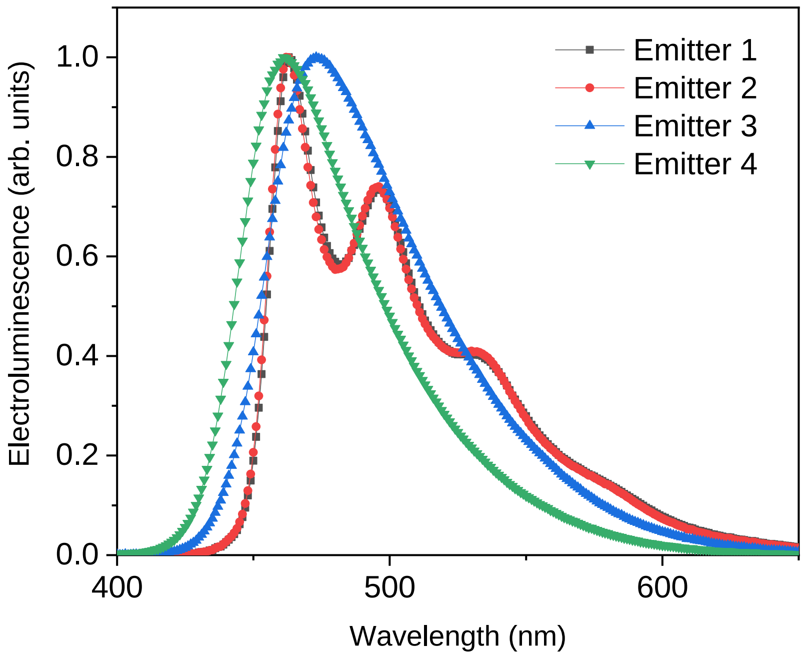

Supplementary Figure S3. Electroluminescence spectra of whole devices.

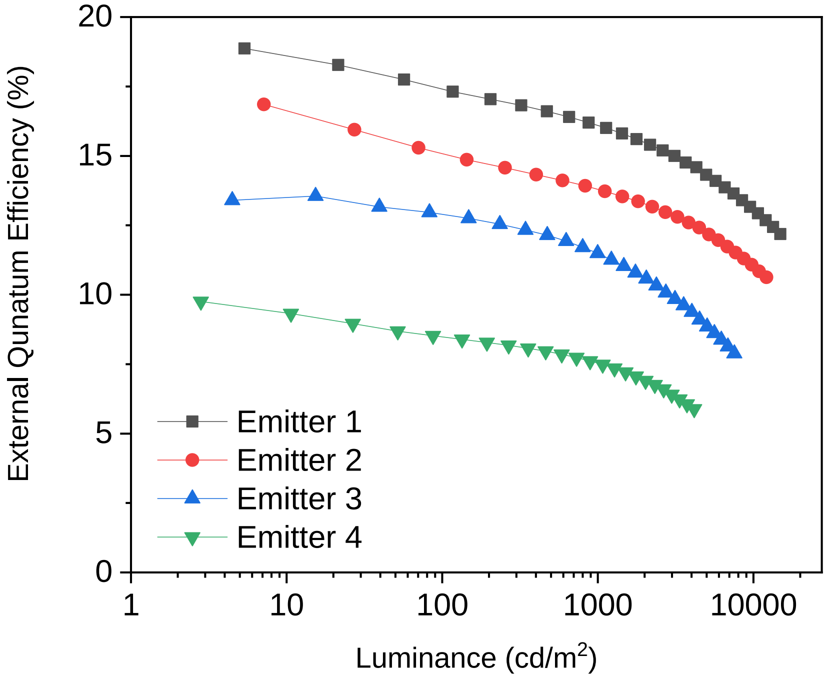

Supplementary Figure S4. The external quantum efficiency of whole devices with respect to the luminance.

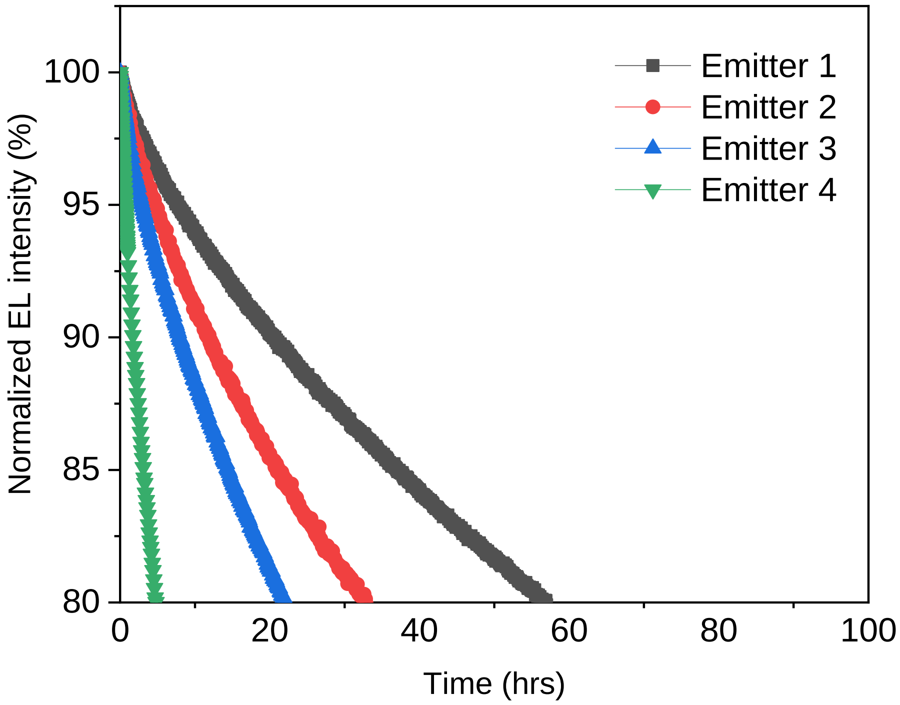

Supplementary Figure S5. Luminance decays during the operation of whole devices in a constant current driving mode.

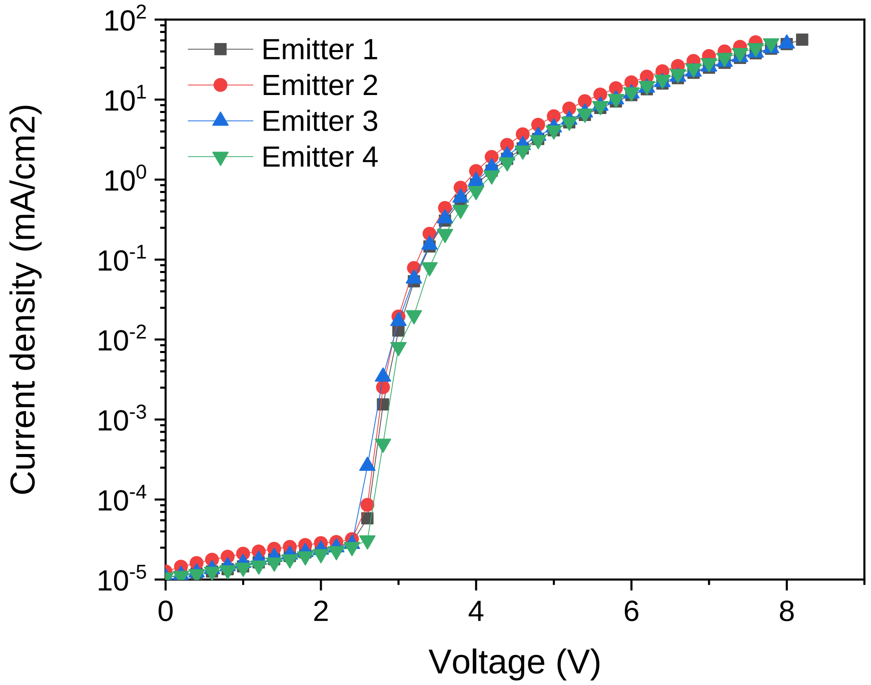

Supplementary Figure S6. The current density-voltage curves of whole devices.

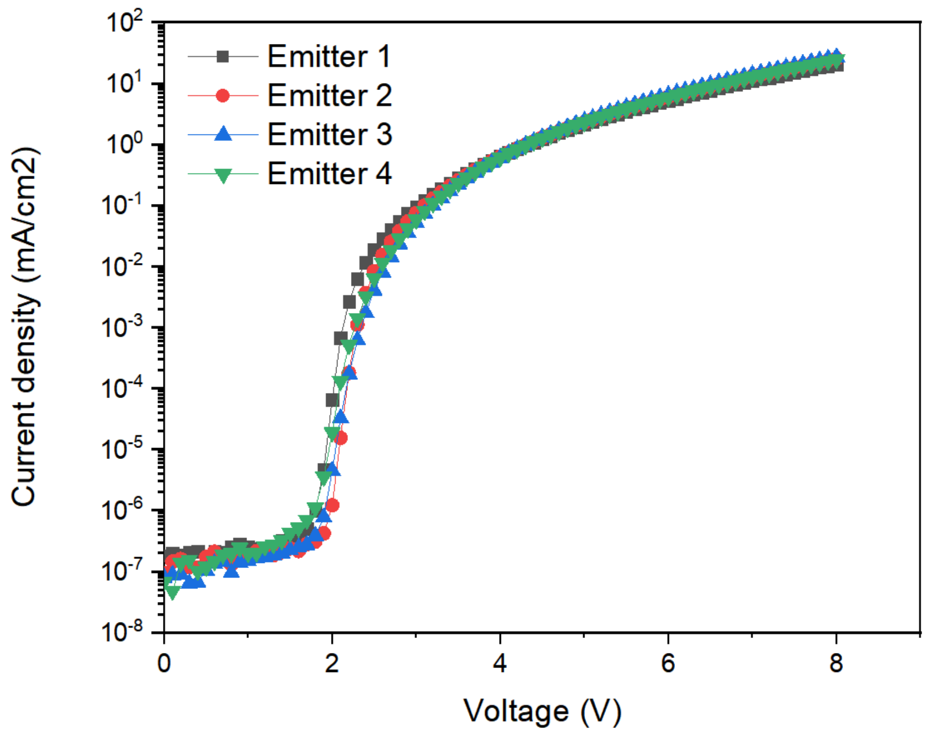

Supplementary Figure S7. The current density-voltage curves of electron-only devices.

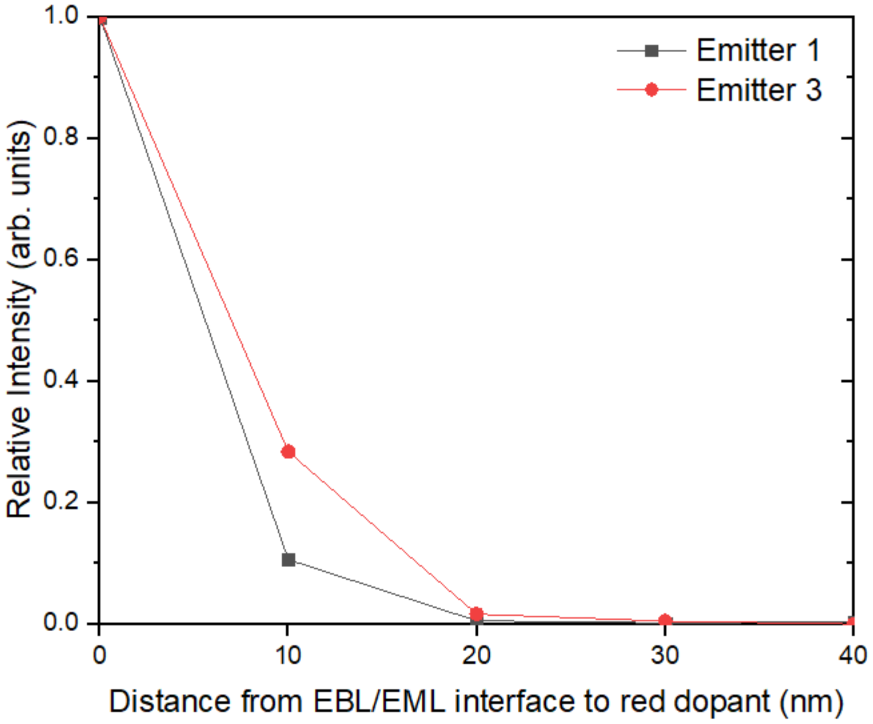

Supplementary Figure S8. Relative electroluminescence intensities of the red dopants of probe devices.

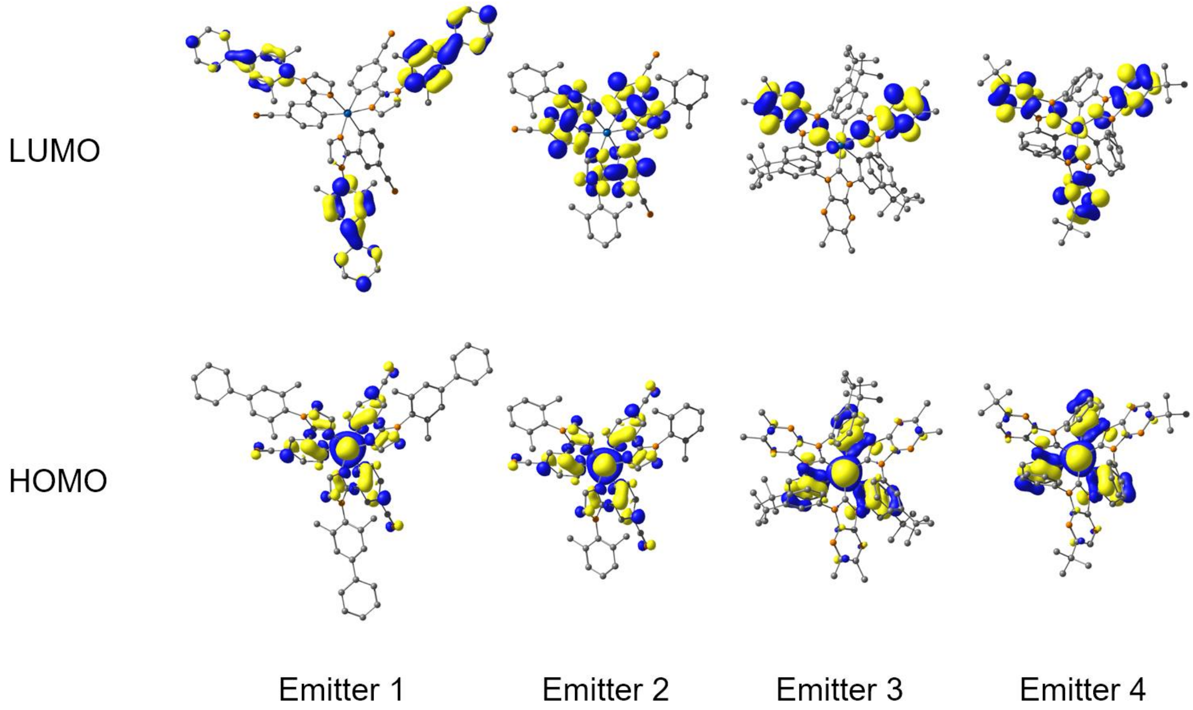

Supplementary Figure S9. Frontier orbitals of neutral emitters 1-4.

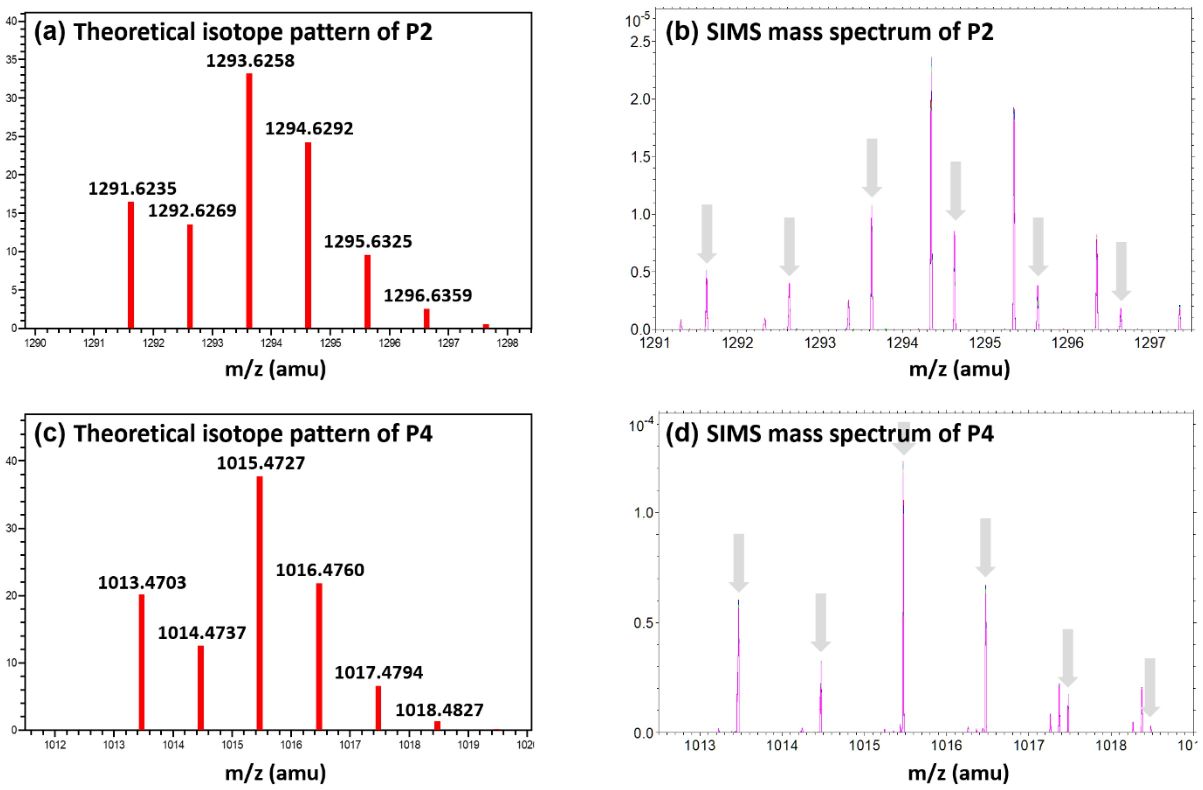

Supplementary Figure S10. Isotope distribution of primary degradation products which includes iridium. (a, c) Calculated theoretical isotope distributions for the primary degradation products P2 (a) and P4 (c). (b, d) Experimentally obtained mass spectra from the emissive layer (EML) of an aged device showing peaks corresponding to the mass-to-charge (m/z) values of degradation products P2 (b) and P4 (d).

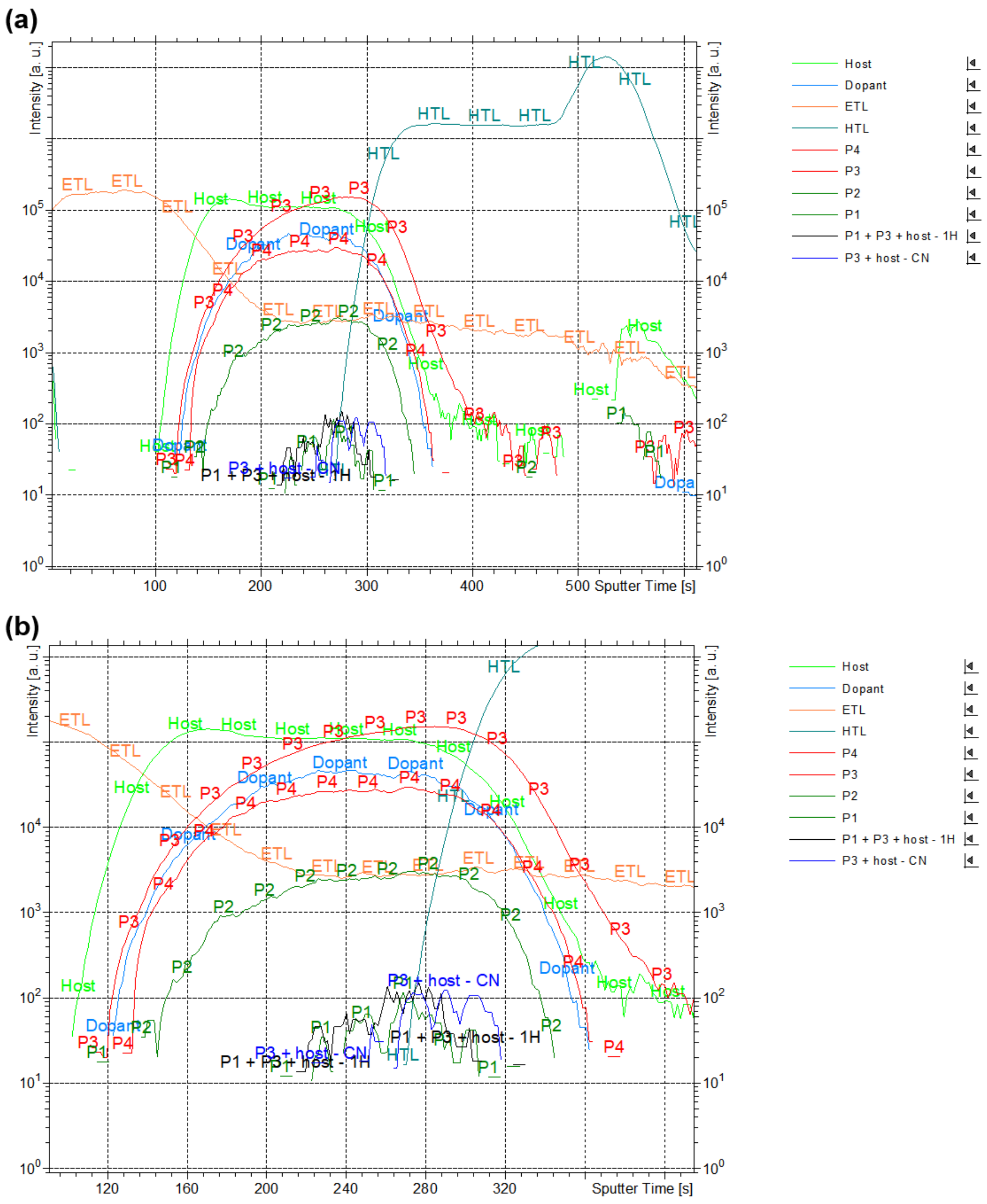

Supplementary Figure S11. Depth profile of the mass spectrum of the aged device whose luminance dropped to 10% of the initial value (Emitter 3) (a) Mass spectrum peak intensity with respect to the ion beam sputter time. (b) Magnified version of (a) for a clearer view of secondary degradation products P1+P3+host-1H

Supplementary Tables

Supplementary Table S1. The photoluminescence quantum yield of emitters measured in film.

	Emitter 1	Emitter 2	Emitter 3	Emitter 4
Photoluminescence quantum yield	0.802	0.777	0.442	0.340

Supplementary Table S2. The performance of whole devices at 500 cd m^{-2}.

	λ_{max} [nm]	CIE (x, y)	Voltage [V]	Max EQE [%]	EQE [%]	Power efficiency [lm W^{-1}]	Current efficiency [cd A^{-1}]	LT95 [hrs]
**Emitter 1**	463	(0.190, 0.330)	4.2	18.9	16.6	27.1	36.5	7.7
**Emitter 2**	462	(0.188, 0.328)	4.1	16.9	14.2	24.0	31.2	4.7
**Emitter 3**	473	(0.161, 0.289)	4.4	13.6	12.1	16.7	23.5	2.9
**Emitter 4**	461	(0.148, 0.178)	5.0	9.8	7.9	7.1	11.2	0.7

Supplementary Table S3. Operational condition of WD

	Emitter 1	Emitter 2	Emitter 3	Emitter 4
Radiance (mW sr^{-1} m^{-2})	2000	2000	2000	2000
Luminance (cd m^{-2})	573	561	487	350
Current (mA)	0.064	0.072	0.079	0.137

Supplementary Table S4. Operational condition of EOD

	Emitter 1	Emitter 2	Emitter 3	Emitter 4
UV lamp intensity (mW)	23.2	22.3	15	18.2
Initial radiance (mW sr^{-1} m^{-2})	19138	18087	8991	9000
Current for exciton+electron EOD (mA)	0.064	0.072	0.079	0.137

Supplementary Table S5. The bond dissociation energy of each degradation process (ΔH, unit: kcal/mol).

Degradation process			Emitter 1	Emitter 2	Emitter 3	Emitter 4	Electronic state
6-coordinated complex, Intra-ligand bond dissociation	Benzylic hydrogen (C-H)		68.20	64.20	-[a]	-[a]	Anion doublet
			26.75	26.48	-[a]	-[a]	Neutral triplet
	C-N bond	**Path 1**	25.49	18.64	24.00	27.66	Anion doublet
		**Path 5**	17.87	17.16	19.16	22.34	Neutral triplet
5Ar intermediate, Intra-ligand bond dissociation	**Path 2** Aryl – N-heterocyclic ring (C-C or C-N)		78.48	78.50	27.26	31.32	Anion doublet
5Ar intermediate, Metal-ligand bond dissociation	**Path 3** Iridium – N-heterocyclic ring (Ir-:N or Ir-:C)		2.84	1.51	4.56	15.11	Anion quartet
3MC intermediate, Metal-ligand bond dissociation	**Path 4** Iridium – Aryl ($Ir-C_{Ar}$)		33.07	35.96	33.27	35.57	Neutral triplet

[a] Emitters 3 and 4 do not have benzylic hydrogen.

Supplementary Table S6. The energy profile for the Ir-ligand bond cleavage reaction. The relative Gibbs free energy is computed as the Gibbs free energy difference between the transition structure and the 6-coordinated emitter (ΔG, unit: kcal/mol).

**Degradation process**		Emitter 1	Emitter 2	Emitter 3	Emitter 4
Neutral $S_0 \rightarrow$ Neutral T_1	0-0 excitation energy	59.22	58.56	57.74	58.50
Neutral $T_1 \rightarrow$ 3MC	Transition state	10.24	10.65	17.30	-[a]
	5-coordinated complex	6.74	7.16	15.81	20.06
Neutral $T_1 \rightarrow$ Triplet 5Ar	Transition state	33.64	34.09	29.90	24.64
	5-coordinated complex	28.20	28.79	23.42	22.60
Anion $D_0 \rightarrow$ Doublet 5Ar	Transition state	60.16	54.12	56.98	58.83
	5-coordinated complex	55.94	50.89	55.76	53.84
Anion $Q_1 \rightarrow$ Quartet 5Ar	Transition state	32.75	46.41	27.35	30.32
	5-coordinated complex	23.88	22.40	27.28	26.03

[a] Barrier energy is too small to detect

Supplementary Table S7. The C-N bond lengths of the 6-coordinated emitters (unit: Å).

	Emitter 1	Emitter 2	Emitter 3	Emitter 4
Neutral	1.439/1.439/1.439	1.440/1.440/1.440	1.436/1.436/1.436	1.434/1.434/1.434
Anion	1.436/1.436/1.436	1.430/1.430/1.430	1.435/1.430/1.428	1.431/1.430/1.430

Supplementary Discussion

Charge and exciton distribution of whole device

We used an electron transport type host molecule to minimize the influence of holes on the lifetime of the whole device (WD). [S1] In the EML of WDs, emitter molecules are doped in the host film. As the HOMO energy of the emitter molecules is higher than that of host molecules, the doped emitter molecules can improve the hole mobility in the EML. This requires a deeper investigation into the polaron distribution to ensure that the effect of the hole is well suppressed in the devices. Hence, we measured the exciton distribution in the EML by using the methodology described in the reference [S2]. We fabricated probe devices with the same structure as the WDs except an inserted thin red dye layer inside the EML. Here, $Ir(dmpq)_2(acac)$[S3] was co-doped at 2 vol% at different positions separated by 10 nm in the EMLs, with a doping layer width of 1 nm. The thin layer emits red light by accepting exciton energy from adjacent excited molecules. Comparing the relative intensity of red light from the probe devices, we measured the exciton densities as a function of the position of the probe layer. The relative intensity is calculated using the following equation.

$$\text{Relative Intensity}(x) = \frac{(\text{Intensity of probe device @ 612 nm})(x) - (\text{Intensity of WD @ 612nm})}{\max\{(\text{Intensity of probe device @ 612 nm})(x)\}}$$

As the charge balances of WDs are similar to each other, we fabricated probe devices for Emitter 1 and 3 as examples. Our data shows that excitons are highly concentrated near the EBL/EML interface and are almost absent in the region farther than 20 nm away from the interface. (Figure S8) The result implies that charge recombination occurs predominantly at the EBL/EML interface, and the holes will be concentrated at the same interface.

Supplementary Method

The distribution of the additional electron on the emitter molecules

The electron density distribution in the emitter molecules is determined by calculating the difference between the atomic charges in their neutral and anionic states. The electron density of each emitter molecule was obtained from its ground state structure using Gaussian 16 [S4] software, and the atomic charge of each atom was calculated using Natural Bond Orbital (NBO) analysis. [S5]

The added electrons occupy the lowest unoccupied molecular orbital (LUMO) of the emitter, which is uniformly distributed across the three ligands (Fig. S9). For iridium-based phosphorescent emitters, the energies of the first three lowest unoccupied molecular orbitals are almost identical and located on each ligand. Thus, a small perturbation can break the symmetry by pushing the electron density toward one of the three ligands. This electronic structure of the iridium-based phosphorescent emitters has been verified by spectroscopic experiments and quantum chemical calculations. [S6, S7] Therefore, the atomic charge changes obtained from each ligand were summed to identify the atoms where the added electrons were located. We used a threshold of 0.01.

Supplementary References

S1. Ihn, S.-G., et al. An Alternative Host Material for Long-Lifespan Blue Organic Light-Emitting Diodes Using Thermally Activated Delayed Fluorescence. *Adv. Sci.* **4**, 1600502 (2017)

S2. Zhang, Y., Lee, J. and Forrest, S. R. Tenfold increase in the lifetime of blue phosphorescent organic light-emitting diodes. *Nat. Commun.* **5**, 1–7 (2014).

S3. Kim, D.H., et al. Highly Efficient Red Phosphorescent Dopants in Organic Light-Emitting Devices. *Adv. Mater.* **23,** 2721-2726 (2011)

S4. Gaussian 16, Revision B.01, Frisch, M. J., et al. Gaussian, Inc., Wallingford CT, (2016)

S5. Glendening, E. D. and Weinhold, F. Pauling's Conceptions of Hybridization and Resonance in Modern Quantum Chemistry. *Molecules* **26**, 4110 (2021).

S6. Hofbeck, T. and Yersin, H. The Triplet State of *fac*-Ir(ppy)3. *Inorg. Chem.* **49**, 9290-9299 (2010).

S7. Liu, T., Xia, B.-H., Zheng, Q.-C., Zhou, X., Pan, Q.-J. and Zhang, H.-X. DFT/TD-DFT investigation on Ir(III) complexes with *N*-heterocyclic carbene ligands: Geometries, electronic structures, absorption, and phosphorescence properties. *J. Comput. Chem.* **31**, 628-638 (2010).
